# Matrix Effects on the Quantification of NanoLuc Bioluminescence in Ex Vivo Organ Homogenates

**DOI:** 10.3390/s26196135

**Published:** 2026-09-28

**Authors:** Roman O. Melikov, Maria O. Samokhina, Maria A. Yurchenko, Alexey V. Orlov, Maxim P. Nikitin

**Affiliations:** 1 Moscow Center for Advanced Studies, 123592 Moscow, Russia; 2 Prokhorov General Physics Institute of the Russian Academy of Sciences, 119991 Moscow, Russia; 3 Department of Nanobiomedicine, Sirius University of Science and Technology, 354340 Sirius, Russia; 4 Shemyakin-Ovchinnikov Institute of Bioorganic Chemistry, Russian Academy of Sciences, 117997 Moscow, Russia

**Keywords:** luciferase, NanoLuc, furimazine, bioluminescence, organ homogenates, ex vivo, matrix effects, limit of detection

## Abstract

Bioluminescent reporters and biosensors are widely used to quantify gene expression, evaluate delivery systems and assess biodistribution because they provide low-background readouts over a broad dynamic range. The NanoLuc–furimazine system is particularly attractive owing to its high brightness and stability. Ex vivo analysis of organ homogenates offers a controlled approach for measuring tissue-associated reporter activity, yet matrix-dependent effects are often overlooked in quantitative interpretation. Here, we systematically evaluated how mouse organ homogenates affect NanoLuc–furimazine measurements by spiking defined amounts of purified NanoLuc into different organ matrices. Across matrices, optical profiles, blank signals, calibration slopes, and estimated limits of detection and quantification varied, resulting in substantial differences in the measured NanoLuc signal. Further analysis showed that NanoLuc-independent furimazine luminescence arises from lysis-buffer components and protein-containing environments. Matrix-matched blank subtraction reduced background-related bias, whereas matrix-specific calibration demonstrated differences in analytical response, particularly for weak signals and optically dense samples. These findings highlight the extent of matrix-dependent variability in ex vivo NanoLuc measurements and support more accurate interpretation of reporter signals in delivery, biodistribution, biosensing, and gene-expression studies.

## 1. Introduction

Modern biomedical research increasingly relies on molecular and nanoscale platforms, including biosensors, delivery systems and theranostic nanoagents, to detect biological cues, deliver functional payloads and generate measurable responses in complex biological environments [1,2,3,4]. Reliable evaluation of these platforms requires quantitative assessment of functional reporter activity, circulation kinetics, biodistribution and accumulation in target tissues using complementary analytical readouts [5,6,7].

Bioluminescent systems are widely used as the basis of optical biosensors and reporter assays for monitoring biological processes in cells, tissues and living organisms [8,9,10]. In these assays, a luciferase enzyme catalyzes oxidation of a small-molecule substrate, generating electronically excited intermediates that emit light upon relaxation to the ground state without external optical excitation. This distinguishes bioluminescence from fluorescence-based approaches and avoids excitation-related autofluorescence, enabling low-background detection with high sensitivity and a broad dynamic range [11]. These properties have supported the development of diverse bioluminescent biosensors for intracellular sensing, protein interaction analysis and enzymatic activity detection [12,13,14]. In delivery studies, luciferase assays are particularly useful for quantifying transgene expression, tissue-associated reporter activity and functional payload release after administration of DNA, mRNA or oligonucleotide-based systems [15,16,17].

Among available luciferase systems, NanoLuc has become one of the most attractive reporters owing to its small size, high stability, ATP-independent reaction and exceptionally bright luminescence with the synthetic imidazopyrazinone substrate furimazine [18]. The NanoLuc–furimazine system was engineered from the catalytic subunit of the deep-sea shrimp *Oplophorus gracilirostris* luciferase and was shown to provide a bright glow-type signal over a broad useful concentration range [19]. Since then, NanoLuc-based technologies have been widely applied to gene expression assays, protein–protein interaction studies, biosensing, cell tracking and in vivo bioluminescence imaging [20,21,22,23].

In vivo bioluminescence imaging is highly informative because it enables non-invasive spatial and temporal visualization of reporter distribution in the same animal over time [24,25]. However, the detected photon flux is not determined by reporter abundance alone. It also depends on local substrate availability, which is influenced by substrate solubility, pharmacokinetics, route of administration, tissue distribution and cellular uptake or efflux, as well as on tissue depth, absorption, scattering and the emission spectrum of the reporter [26,27,28]. These limitations are particularly relevant for blue-emitting reporters such as NanoLuc–furimazine and have motivated the development of improved NanoLuc substrates with enhanced solubility, bioavailability or in vivo imaging performance, including hydrofurimazine and fluorofurimazine [29,30]. Thus, the quantitative strength of in vivo bioluminescence imaging lies primarily in longitudinal measurements performed under standardized conditions, whereas photon flux cannot be interpreted as a direct measure of reporter abundance when different organs are compared. Ex vivo imaging of isolated organs reduces attenuation by overlying tissues and allows a more direct comparison of tissue-associated signals. Nevertheless, the measured signal remains dependent on prior substrate delivery and distribution, as well as on the geometry and optical properties of the isolated tissue.

Ex vivo analysis of organ homogenates can reduce some of these uncertainties and provide a more controlled approach to quantifying tissue-associated reporter activity. Homogenization disrupts intact tissue architecture, enables uniform substrate addition under defined reaction conditions, improves sample averaging and reduces the influence of organ size, geometry and source depth on detected light output. Accordingly, luciferase activity in tissue homogenates is frequently used as a terminal quantitative endpoint in studies of nucleic acid delivery and biodistribution, including direct NanoLuc measurements in homogenized organs [6,23,31]. Nevertheless, homogenates remain optically and chemically complex matrices. They contain proteins, lipids, chromophores, blood-derived components and particulate material, all of which can affect substrate background, reporter activity or detected light output. Earlier work with luciferase assays showed that hemoglobin in tissue samples can mask reporter activity and lead to underestimation of luciferase expression [32]. More generally, mammalian tissue absorption and scattering strongly influence the sensitivity of bioluminescence detection, especially for shorter-wavelength emission [26].

A second source of complexity arises from the environment-sensitive chemistry of coelenterazine-type substrates. Although furimazine was developed as an optimized NanoLuc substrate, structurally related imidazopyrazinone compounds are known to undergo enzyme-independent luminescent reactions that depend on the surrounding physicochemical environment [33]. Albumin has been reported to support coelenterazine chemiluminescence, and synthetic coelenterazine indicators have even been developed for luminescent detection of human and bovine serum albumins [34,35,36]. These observations suggest that serum- or albumin-containing samples may generate a substrate-dependent background that is not equivalent to enzymatic NanoLuc emission. Such effects may be negligible at high reporter abundance, but they can become important when NanoLuc concentrations approach the lower detection range.

Despite the broad use of NanoLuc as a sensitive reporter, systematic matrix-aware validation of NanoLuc–furimazine measurements in ex vivo organ homogenates remains limited. In delivery and biodistribution studies, luciferase activity is commonly reported as raw luminescence or as luminescence normalized to tissue mass or total protein content [37,38,39]. Such measurements provide a practical estimate of organ-associated reporter activity but do not necessarily account for matrix-matched substrate background, matrix-dependent response or assay-specific detection and quantification limits. Consequently, equal reporter activity cannot be assumed a priori to generate an equivalent analytical response in different organ lysates. These effects are particularly relevant at low reporter abundance, where background might represent a larger fraction of the measured signal.

To improve the interpretation of tissue-associated NanoLuc activity in biological studies, we systematically evaluated how ex vivo organ homogenates influence the analytical response of the NanoLuc–furimazine assay. In this framework, organ homogenates served as biological sample matrices and purified NanoLuc as the reporter analyte. Spiking defined amounts of NanoLuc into the lysates allowed us to hold reporter input constant and compare matrix-dependent effects under controlled conditions. We characterized matrix optical properties, protein content, furimazine-dependent background, signal attenuation, and calibration behavior, and estimated matrix-specific limits of detection (LOD) and quantification (LOQ). We further examined NanoLuc-independent furimazine luminescence in serum, purified-protein, and lysis-buffer conditions. Together, these analyses provide a controlled assessment of matrix-dependent variability in NanoLuc measurements and identify analytical factors that should be considered when NanoLuc activity is quantitatively compared across tissue-derived samples.

## 2. Materials and Methods

### 2.1. Reagents

The following reagents and assay kits were used in this study: furimazine (FMZ; Abisense, Sirius, Russia), coelenterazine h (CTZ-h; Nanolight Technology, Pinetop, AZ, USA), Tris (Suzhou Yacoo Science Co., Suzhou, China), Tris-HCl (Suzhou Yacoo Science Co., Suzhou, China), dimethyl sulfoxide (DMSO; Expresolv, Bavla, Gujarat, India), Nonidet P-40 substitute (NP-40; Servicebio, Wuhan, China), Triton X-100 (Servicebio, Wuhan, China), glycerol (Panreac AppliChem, Darmstadt, Germany), sodium chloride (NaCl; Lenreactiv, St. Petersburg, Russia), hemoglobin (Worthington Biochemical Corp., Lakewood, NJ, USA), ovalbumin (Worthington Biochemical Corp., Lakewood, NJ, USA), fetal bovine serum (FBS; Cell Technologies, Paneco, Moscow, Russia), bovine serum albumin (BSA; Diaem, Moscow, Russia), human normal immunoglobulins (IgG; Microgen, Moscow, Russia), bicinchoninic acid (BCA) protein assay kit (Abisense, Sirius, Russia), Zoletil 100 (Virbac, Carros, France), Xyla (Interchemie Werken De Adelaar Eesti AS, Püünsi, Estonia), and IPTG (BLDpharm, Shanghai, China). IgG was dialyzed against distilled water before use. All other reagents were used as received. The standard lysis buffer contained 20 mM Tris-HCl (pH 7.5), 150 mM NaCl, 1% NP-40 and 5% glycerol. Stock solutions of FMZ and CTZ-h were prepared in DMSO and ethanol, respectively, and diluted in phosphate-buffered saline (PBS) to the required working concentrations immediately before each experiment.

### 2.2. NanoLuc Expression and Purification

Recombinant NanoLuc luciferase was expressed in *E. coli* BL21(DE3) cells transformed with a pET-21a-based NanoLuc expression plasmid carrying an ampicillin-resistance marker. Cells were grown in 2 × YT medium at 37 °C to an OD600 of approximately 0.6, induced with 1 mM IPTG and further incubated overnight at 18 °C. Cell pellets were resuspended in a buffer containing 20 mM Tris-HCl, 300 mM NaCl, 1 mM PMSF and 0.5 mg/mL lysozyme, pH 8.0, and disrupted by sonication on ice. After clarification by centrifugation, recombinant His-tagged NanoLuc was purified from the soluble fraction by Ni^2+^-NTA affinity chromatography and eluted with imidazole-containing buffer. The purified protein was dialyzed against 50 mM Tris-HCl and 150 mM NaCl (pH 7.5) and filtered through a 0.22 µm membrane. The concentration of purified NanoLuc was determined spectrophotometrically by measuring absorbance at 280 nm, and its purity was assessed by SDS–PAGE. NanoLuc was aliquoted to avoid repeated freeze–thaw cycles and stored at −20 °C in the presence of 20% glycerol and 1 mM DTT. Within each experiment, all matrices were measured in parallel using NanoLuc dilutions prepared from the same stock solution, which was kept at 4 °C throughout the experiment. Matrix-specific calibration curves were generated within each experiment to account for potential day-to-day or aliquot-to-aliquot variation in NanoLuc activity.

### 2.3. Animals and Sample Collection

Male BALB/c mice weighing 24–26 g and aged 2–4 months were used in the study. For experimental manipulations, animals were anesthetized by intraperitoneal injection of Zoletil 100/Xyla at doses of 40/1.5 mg/kg. To obtain tissue samples, mice were euthanized by cervical dislocation, and blood, heart, lungs, liver, spleen and kidneys were collected for ex vivo analysis. Animals were not transcardially perfused before organ collection. After excision, the organs were briefly rinsed in ice-cold 0.9% NaCl solution and gently blotted to remove excess surface liquid before weighing and homogenization. All procedures were approved by the Institutional Animal Care and Use Committee of the Shemyakin-Ovchinnikov Institute of Bioorganic Chemistry, Russian Academy of Sciences, according to protocol no. 375/2023, approved for the period from 20 September 2023 to 19 September 2026.

### 2.4. Preparation of Organ Homogenates

Unless otherwise stated, a 100 mg piece of solid organ, or 100 µL of blood, was placed into 1.5 mL tubes containing metal beads and 1 mL of lysis buffer. Samples were homogenized using a FastPrep-24 5G sample disruption instrument (MP Biomedicals, Solon, OH, USA) at 6 m/s for three 40 s cycles and then clarified by centrifugation at 15,000× *g* for 10 min at 4 °C. The supernatants were collected and used as the organ lysate. For tissue-load experiments, liver samples of 10, 50, 100 and 200 mg were homogenized in 1 mL of lysis buffer using the same procedure. Clarified lysates were analyzed immediately after preparation.

### 2.5. Absorbance Spectroscopy and Hemoglobin-Equivalent Estimation

Absorbance spectra of clarified lysates were recorded using a CLARIOstar Plus multimodal plate reader (BMG Labtech, Ortenberg, Germany). For each measurement, 100 µL of lysate was added to a 96-well plate, and absorbance spectra were acquired from 340 to 700 nm with a 1 nm step. The blood sample was additionally diluted 10-fold before spectral acquisition to avoid detector overflow. Hemoglobin-associated optical burden was estimated using absorbance at 414 nm and a calibration curve prepared from hemoglobin standards. Because hemoglobin absorbance depends on oxidation state, sample clarification and matrix composition, these values were interpreted as hemoglobin-equivalent estimates rather than absolute clinical hemoglobin concentrations.

### 2.6. Protein Quantification

Total protein concentration in clarified lysates was measured using a BCA protein assay kit with BSA standards. Lysate samples were diluted 10-fold before analysis. For each measurement, 10 µL of the diluted sample or standard was mixed with 100 µL of BCA working reagent according to the manufacturer’s instructions. After 30 min of incubation, absorbance at 562 nm was measured using a CLARIOstar Plus multimodal plate reader. Protein concentration was calculated from the BSA calibration curve.

### 2.7. Luminescence Measurements

Luminescence measurements were performed using a CLARIOstar Plus multimodal plate reader in kinetic mode in black 96-well plates without an emission filter. The focal height was set to 11 mm and the gain to 3600, and measurements were performed at 23 °C. Unless otherwise stated, 100 µL of sample was mixed with 100 µL of 100 µM FMZ solution and briefly pipetted. Luminescence recording was initiated 2 min after substrate addition, with no additional shaking applied during measurement. Luminescence kinetics were recorded for 20 min with measurements taken every 2 min. Each individual measurement was acquired using an integration time of 0.8 s. The kinetic area under the curve (AUC) over the acquisition window was calculated using the trapezoidal rule and used as the primary luminescence readout throughout the study. For luminescence values *L_i_* expressed in relative light units (RLU) and recorded at time points *t_i_*, the AUC was calculated as

(1) $$AUC=\sum_{i=1}^{n-1}\frac{L_{i}+L_{i+1}}{2}\left(t_{i+1}-t_{i}\right).$$

where *n* is the number of measurements, time is expressed in minutes, and AUC is expressed in RLU·min. *n* = 11 in the present study. Because successive measurements were separated by a constant interval of 2 min, Equation (1) reduces to

(2) $$AUC=2\left[\frac{L_{1}+L_{11}}{2}+\sum_{i=2}^{10}L_{i}\right]=L_{1}+2\sum_{i=2}^{10}L_{i}+L_{11}.$$

Unless otherwise indicated, all samples were measured at a final furimazine concentration of 50 µM in the assay well.

### 2.8. NanoLuc Spike-In Experiments in Clarified Lysates

Purified NanoLuc was serially diluted in PBS supplemented with 0.01% BSA and spiked into lysis buffer or clarified organ lysates before substrate addition. In a typical spike-in experiment, 10 µL of NanoLuc solution at concentrations ranging from 5000 to 5 fM was added to 90 µL of lysis buffer or organ lysate, followed by addition of 100 µL of 100 µM furimazine working solution. The final assay volume was 200 µL, resulting in final NanoLuc concentrations of 250, 50, 25, 5, 2.5, 0.5 and 0.25 fM in the assay well. Unless otherwise stated, all protein, serum and substrate concentrations reported in the text, figures and calibration analyses refer to the final concentration in the assay well. Matrix blanks were prepared in the same format by adding 10 µL of PBS containing 0.01% BSA, but no NanoLuc, to 90 µL of the corresponding lysis buffer or organ matrix, followed by addition of furimazine. This corresponded to a final BSA concentration of 0.0005% in both NanoLuc-containing samples and their matrix-matched blanks. For the matrix-aware signal interpretation, NanoLuc was spiked into organ matrices at final concentrations of 40 and 8 fM and analyzed as raw AUC, blank-corrected AUC and matrix-calibrated concentration equivalent.

### 2.9. Pre-Homogenization NanoLuc Spike-In Experiments

To evaluate the influence of tissue processing on the detectable NanoLuc signal, purified NanoLuc was added directly to tissue samples before homogenization. For the organ-comparison experiment, 100 mg of each solid organ or 100 µL of blood was mixed with 1 mL of lysis buffer. NanoLuc was added before tissue disruption at concentrations of 500, 50 or 5 fM in the homogenization mixture. Samples were then homogenized and clarified using the previously described procedure. Assuming complete recovery, these inputs corresponded to nominal final assay concentrations of 250, 25 and 2.5 fM after mixing the clarified lysate 1:1 with the furimazine working solution.

In the liver tissue-load experiment, NanoLuc was added before homogenization to liver samples of 10, 50, 100 or 200 mg placed in 1 mL of lysis buffer. The NanoLuc input was scaled proportionally to tissue mass to maintain the same nominal reporter input per unit tissue mass. NanoLuc concentrations in the homogenization mixture were 50, 5 and 0.5 fM for 10 mg samples; 250, 25 and 2.5 fM for 50 mg samples; 500, 50 and 5 fM for 100 mg samples; and 1000, 100 and 10 fM for 200 mg samples. After homogenization and clarification, 100 µL of lysate was analyzed in the standard 200 µL assay format. After the 1:1 dilution with furimazine working solution, the nominal final NanoLuc inputs normalized to the starting tissue mass were 2.5, 0.25 and 0.025 fM/mg for all tissue loads.

### 2.10. Luminescence Emission Spectra

Luminescence emission spectra were recorded for samples containing furimazine in the presence of 1 pM NanoLuc, 50 g/L BSA or 50% FBS. Spectra were acquired from 340 to 600 nm with a 2 nm step using the spectral scanning mode of the plate reader, with a spectral bandwidth of 40 nm. Spectra were background-corrected and normalized for comparison of spectral shape. PBS with furimazine alone was measured under the same conditions as a substrate/instrument control.

### 2.11. NanoLuc-Independent Luminescence Experiments

NanoLuc-independent luminescence was evaluated in serum, purified-protein and lysis-buffer conditions in the absence of NanoLuc. FBS and mouse serum were tested at 50%, 5% and 0.5% final concentrations in the presence of 50 µM FMZ or CTZ-h. For comparison of purified proteins, BSA, IgG and ovalbumin were tested at final concentrations of 50, 12.5 and 0.5 µM. NanoLuc was included as an enzymatic positive control at a final concentration of 0.5 µM.

BSA-dependent FMZ luminescence was further assessed by titrating either BSA or FMZ concentration. For the BSA titration, the final FMZ concentration was fixed at 50 µM, while the final BSA concentration was varied from 50 to 0.00064 g/L. For the FMZ titration, the final BSA concentration was fixed at 25 g/L, while the final FMZ concentration was varied from 200 to 0.01 µM by serial dilution. The DMSO percentage was kept constant across the FMZ dilution series. To evaluate the effect of lysis buffer composition, individual buffer components and nonionic detergents, including NP-40, Triton X-100, Tween-20 and Tween-80, were tested under matched substrate conditions.

### 2.12. Data Analysis and Statistics

All experiments involving organ lysates were performed using samples from at least three independent animals; throughout the manuscript, *n* denotes the number of biological replicates. Each biological replicate corresponded to an organ sample from an individual mouse. Technical replicates represented separate measurements of samples derived from the same mouse. Technical replicates were averaged within each animal and concentration before curve fitting and statistical analysis and were not treated as independent observations. Separate matrix- and tissue-load-specific calibration curves were fitted for samples from six independent mice using weighted least-squares linear regression of raw AUC against final NanoLuc concentration. A weighting factor of $w=1/{AUC}^{2}$ was applied to account for increasing residual variance at higher signal intensities and to prevent high-response observations from having disproportionate influence.

Raw AUC, blank-corrected AUC and calibration-derived concentration equivalents were treated as parallel analytical representations. The blank-corrected response was calculated as

AUC_corrected_ = AUC_raw_ − MEAN(AUC_blank_). (3)

For an uncorrected matrix-specific calibration

AUC_raw_ = *kC* + *b*, (4)

where *C* is the final NanoLuc concentration, *k* is the slope and *b* is the Y-intercept. The concentration equivalent was calculated as

*C*_estimated_ = (AUC_raw_ − *b*)/*k*. (5)

Matrix-matched blank distributions were obtained from 12 independent mice. LOD and LOQ values were estimated using a blank/calibration-curve standard-deviation method (the 3σ/10σ approach) [40,41]. The LOD and LOQ signal thresholds were defined as

AUC_LOD_ = MEAN(AUC_blank_) + 3 SD(AUC_blank_), (6)

AUC_LOQ_ = MEAN(AUC_blank_) + 10 SD(AUC_blank_). (7)

These thresholds were converted to NanoLuc concentrations using each of the six individual calibration equations, as described in Equation (5). The resulting six calibration-derived estimates were used to calculate the mean estimated LOD and LOQ values, as well as their SDs and 95% confidence intervals.

Comparisons among organ-derived matrices obtained from the same animals were performed using repeated-measures (RM) one-way ANOVA with the Geisser–Greenhouse correction. Exact *p*-values are reported where relevant, and *p* < 0.05 was considered statistically significant.

## 3. Results and Discussion

### 3.1. Effect of Organ Matrix on NanoLuc Detection

To evaluate how tissue matrices influence ex vivo bioluminescence measurements, organ homogenates were prepared under standardized conditions. Mouse heart, lung, liver, spleen, and kidney samples were homogenized in lysis buffer at a tissue-to-buffer ratio of 100 mg/mL and clarified by centrifugation. Blood samples were mixed with lysis buffer at 100 µL per 1 mL ratio and processed in parallel. The resulting matrices were analyzed by absorbance spectroscopy and luminescence assays. To integrate the time-dependent behavior of both the enzymatic NanoLuc signal and matrix-associated background (Figure S1), luminescence was recorded kinetically for 20 min and reported as the area under the curve over the acquisition window.

First, we characterized the optical properties of the matrices. Absorbance spectra acquired from 340 to 700 nm revealed pronounced matrix-specific profiles (Figure 1a). The spectra of organ-derived matrices contained characteristic features in the hemoglobin absorption range, including a pronounced band near 414 nm, consistent with the presence of residual blood-derived hemoglobin (Figure S2a). Hemoglobin-associated optical burden was therefore estimated from absorbance at 414 nm and expressed as an A414-derived hemoglobin-equivalent metric (Figure 1c and Figure S2b). The highest values were observed in blood and, among solid organs, in lung and spleen homogenates. Tissue homogenates also showed a nonspecific elevation of the absorbance spectrum across a broad wavelength range, consistent with light scattering and accumulation of endogenous matrix components. These optical effects may additionally influence quantitative luminescence measurements.

We then evaluated how organ matrices affect the apparent NanoLuc readout by spiking purified NanoLuc into each matrix at final concentrations of 250, 25 and 2.5 fM (Figure 1b). NanoLuc was diluted in PBS containing 0.01% BSA to stabilize the highly dilute enzyme solutions and minimize adsorption-related losses. As an initial descriptive comparison, the raw AUC values obtained in each biological matrix were normalized to the signal measured at the same NanoLuc concentration in lysis buffer, which served as a non-biological reference condition. This representation illustrates how organ-associated signals may appear when interpreted directly, before correction for matrix-specific background.

At the highest tested concentration, signals in solid-organ homogenates were generally close to the lysis buffer response, reaching approximately 90%. RM one-way ANOVA did not reveal a significant overall difference among the solid-organ matrices (*p* = 0.2054). In contrast, blood retained only about 15–20% of the lysis buffer signal across the tested concentration range, consistent with its high optical density and hemoglobin-associated absorbance. At 2.5 fM, signals in solid-organ homogenates corresponded to approximately 28–36% of the lysis buffer response, and the apparent differences among matrices became more pronounced. At this concentration, where matrix-associated background becomes comparable to reporter-derived signal, RM one-way ANOVA revealed statistically significant differences among solid-organ-derived matrices (*p* = 0.0312), indicating that raw normalized comparisons were more strongly influenced by matrix-specific background and intercept effects near the lower signal range. Accordingly, raw inter-organ signal differences should not be interpreted directly as proportional differences in NanoLuc abundance.

However, normalization to the lysis-buffer signal provides only an initial descriptive comparison because it cannot distinguish a reduced reporter response from differences in NanoLuc-independent background. This limitation becomes particularly important at low NanoLuc concentrations, where background constitutes a larger fraction of the total signal. We therefore next quantified NanoLuc-independent luminescence in the corresponding matrix-matched blanks and examined its relationship with the protein content of each matrix.

Total protein concentration and furimazine-dependent blank signal differed among tested matrices (Figure 1d,e). The profile of matrix-associated blank luminescence broadly followed the extracted protein concentration profile, suggesting that protein-rich matrices can support NanoLuc-independent furimazine luminescence. Among the tested solid-organ matrices, liver homogenate showed the highest mean blank signal, consistent with a possible contribution from albumin and other protein environments. This interpretation is consistent with previous reports showing that various protein environments can promote luminescent reactions of coelenterazine-type substrates [34,35,36]. Blood represented an exception: despite its high biological complexity, its measured blank signal was relatively low, potentially reflecting suppression of emitted light in this optically dense matrix. Thus, the measured blank represents a combined contribution from assay components, matrix-supported substrate luminescence and matrix-dependent signal attenuation.

Because NanoLuc was diluted in PBS containing 0.01% BSA, we additionally compared NanoLuc-free lysis buffer, liver and blood lysates supplemented with identical volumes of PBS with or without BSA. BSA markedly increased furimazine-dependent luminescence in lysis buffer but had only minor effects in the biological matrices (Figure S3), where the same albumin amount represented a small addition to endogenous proteins and its contribution was superimposed on matrix-dependent attenuation. Importantly, lysis buffer retained the highest blank signal even without BSA, indicating that signal-attenuating matrix effects predominated under the tested conditions. The high lysis-buffer background therefore further limits raw normalization to this reference, particularly at low NanoLuc concentrations.

Matrix-specific calibration curves were then generated by titrating purified NanoLuc into lysis buffer or organ homogenates (Figure 2a and Figure S4). The response remained linear over the tested concentration range, but the slopes differed among matrices (Table S1, Figure S5). All organ-derived matrices showed lower slopes than lysis buffer, indicating reduced apparent NanoLuc–furimazine response in biological matrices. The smallest slope was observed for blood, followed by lungs, whereas heart showed the highest organ-matrix slope and was closest to lysis buffer.

Organ matrices showed distinct optical extinction across the NanoLuc emission range, including hemoglobin-associated absorption, consistent with an optical contribution to the reduced analytical response. However, the spike-in design does not separate attenuation of emitted light from matrix-dependent changes in the NanoLuc–furimazine reaction. Biomolecular reactions are known to depend strongly on various physicochemical conditions [42]. Accordingly, components of organ lysates could affect furimazine availability or NanoLuc activity by altering the local reaction environment or interacting with assay components. Matrix-specific calibration behavior should therefore be interpreted as an integrated analytical response rather than as evidence for a particular optical or biochemical mechanism. Quantitative separation of these contributions would require an orthogonal optical control, such as a spectrally matched external light source transmitted through, but chemically isolated from, the matrix.

Matrix-matched blank subtraction and matrix-specific calibration address different components of the observed matrix effect. Blank subtraction reduces additive offsets associated with the matrix and assay components, whereas calibration accounts for differences in analytical response reflected by the calibration slopes. Although background may not be strictly additive in complex lysates, blank subtraction substantially improved comparability among the tested solid-organ matrices. At 2.5 fM NanoLuc, the overall difference among solid-organ homogenates was no longer statistically significant after blank subtraction (*p* = 0.0547). However, when blood was included in the analysis, the overall difference remained significant (*p* = 0.0002), consistent with its markedly lower calibration slope. Thus, under the tested conditions, matrix-matched blank subtraction substantially improved low-concentration comparisons among solid-organ homogenates with relatively similar calibration slopes. Matrix-specific calibration remained particularly important for comparisons involving optically dense matrices and for conversion of luminescence signals into reporter concentrations (Figure S6).

Matrix-specific LOD and LOQ values were derived from the corresponding biological blank distributions and calibration curves (Figure 1e; Table S1). Among solid-organ matrices, estimated LOD ranged from 0.35 to 0.57 fM and LOQ from 1.11 to 1.79 fM in the assay well. For blood, the corresponding values were 1.03 and 3.11 fM, whereas for lysis buffer they were 0.77 and 2.22 fM (Figure 2b,c). Despite the lower calibration slopes observed in organ matrices, their lower blank variability partially offset the reduction in analytical sensitivity. Consequently, estimated LOD and LOQ were determined by the balance between calibration slope and blank dispersion rather than by signal attenuation alone.

To assess processing-related losses, NanoLuc was added to solid-organ samples before homogenization and compared with post-lysis spike-in at matched nominal reporter inputs (Figure S7). Pre-homogenization addition generally yielded lower and more variable blank-corrected signals, particularly at the lowest NanoLuc input, although no consistent organ-specific pattern was observed. This comparison suggests that part of the detectable reporter activity may be lost during sample preparation through partial inactivation of NanoLuc during homogenization and/or retention of the enzyme on insoluble tissue debris removed during clarification. These effects are expected to depend on the extraction protocol and may be reduced by optimizing tissue disruption, buffer composition, processing time and temperature, and recovery of NanoLuc from the homogenate.

Residual blood represents another potentially modifiable source of matrix interference. Transcardiac perfusion or more extensive tissue washing may reduce hemoglobin-associated absorption and improve optical conditions [32]. However, a higher signal after blood removal would indicate reduced attenuation but would not necessarily produce a proportional improvement in LOD or LOQ, because these parameters depend on both calibration sensitivity and blank variability. The calibration parameters reported here therefore apply specifically to the non-perfused sample-collection protocol used in this study. Moreover, perfusion would address only the contribution of residual blood and would not eliminate other properties intrinsic to tissue homogenates, including broad absorption and scattering, furimazine-associated background, and biochemical differences. Similarly, more efficient homogenization and recovery of the soluble fraction may reduce reporter inactivation or retention on tissue debris, but cannot remove the intrinsic matrix effects observed in clarified lysates. Optimization of sample preparation should therefore be combined with matrix-matched blank subtraction and, where necessary, matrix-specific calibration.

### 3.2. Effect of Tissue Load on NanoLuc–Furimazine Assay Performance

We next examined how tissue input during homogenization affects NanoLuc–furimazine detection. Liver was selected as a model organ because it provides sufficient material for testing a broad range of tissue-to-buffer ratios and is routinely evaluated in studies of systemically administered nanoparticles and nucleic acid delivery systems, either as the intended target or as a major off-target site in extrahepatic delivery strategies [7,23,43,44]. Liver samples of 10, 50, 100 and 200 mg were homogenized in 1 mL of lysis buffer, and the clarified lysates were analyzed using the same optical and luminescence readouts.

Increasing liver input produced a progressive increase in absorbance across the visible range, consistent with higher matrix density, light scattering and accumulation of endogenous chromophores (Figure 3a). The A414-derived hemoglobin-equivalent metric increased with tissue input, whereas matrix-associated furimazine background varied non-monotonically among the tested tissue loads (Figure S8). Thus, increasing tissue input altered both the optical properties and NanoLuc-independent background of the resulting lysates.

After subtraction of the corresponding tissue-load-matched blanks, the apparent NanoLuc–furimazine response decreased progressively with increasing tissue input across the tested NanoLuc concentrations of 2.5–250 fM (Figure 3b). Thus, increasing tissue load produced a matrix-dependent reduction in the measured NanoLuc response even after correction for substrate-associated background. This effect was also reflected in the calibration slopes: increasing tissue input reduced the slope from approximately 1500 AUC/fM for the 10 mg homogenate to approximately 1000 AUC/fM for the 200 mg homogenate (Figure S9, Table S2). Therefore, tissue load can substantially affect quantitative conversion of luminescence signal into NanoLuc concentration if the same calibration relationship is applied across differently concentrated homogenates. Because increasing tissue input simultaneously changes both the optical density and biochemical composition of the lysate, the tissue-load series demonstrates a concentration-dependent matrix effect but does not identify the specific mechanism responsible for it.

Estimated LOD ranged from 0.36 to 0.68 fM, whereas LOQ ranged from 1.11 to 2.29 fM. Both parameters changed non-monotonically with tissue input: the lowest values were obtained for the 100 mg condition, whereas the highest values were observed for the 200 mg homogenate (Figure 3c,d). Thus, the reduction in calibration slope at higher tissue loads did not translate directly into a proportional increase in LOD or LOQ, because the resulting thresholds depended on the combined effects of calibration sensitivity and blank variability.

Total protein concentration in the clarified lysates increased approximately in proportion to liver tissue input (Figure 3e), indicating that bulk soluble protein recovery did not show an evident decline or saturation over the tested range. Accordingly, for a reporter distributed uniformly throughout the tissue, increasing tissue input would be expected to increase the total amount of NanoLuc available for detection, despite the lower analytical response observed in denser homogenates.

To model uniform reporter abundance per unit tissue, NanoLuc was added before homogenization at inputs scaled proportionally to liver mass (Figure S10). At the two higher reporter levels, larger tissue inputs generally produced higher total blank-corrected signals, indicating that the gain in reporter amount outweighed the reduction in analytical response associated with denser homogenates. At the lowest reporter level, the 10 mg sample yielded a non-positive blank-corrected signal, whereas the corresponding reporter input remained detectable in the larger tissue samples. However, the relationship among the 50, 100 and 200 mg samples became more variable near the detection limit.

Taken together, the organ-comparison and tissue-load experiments show that quantitative ex vivo NanoLuc measurements depend on both matrix composition and the amount of tissue processed. Matrix-matched blank subtraction substantially improved low-concentration comparisons among solid-organ homogenates with relatively similar calibration slopes, whereas matrix-specific calibration remained important for optically dense samples and for conversion of luminescence into reporter concentration. Tissue load introduced an additional trade-off: increasing the amount of tissue reduced the analytical response per unit of NanoLuc but could increase the total detectable signal when reporter abundance scaled with tissue mass.

The controlled spike-in design used purified active NanoLuc and therefore isolated matrix- and processing-related effects under conditions of known reporter input. In biological delivery or gene-expression experiments, the measured activity will additionally depend on delivery efficiency, transcription and translation, intracellular localization, reporter degradation, release during lysis, and retention or loss during clarification. Reporter distribution may also be spatially heterogeneous within an organ; consequently, a small tissue fragment may not accurately represent total organ-associated activity. Matrix calibration can account for the analytical response of the resulting lysate, but it cannot correct these biological and sampling sources of variability.

Several practical approaches can reduce these uncertainties. When feasible, homogenization of the entire organ provides the most complete spatial averaging. A constant tissue-to-buffer ratio should then be maintained; when this is impractical, tissue-load-matched blanks and calibration curves should be generated for the actual homogenization conditions. When whole-organ processing is not feasible, sampling multiple predefined regions and pooling them before homogenization, or analyzing them separately, can reduce bias caused by uneven reporter distribution. Perfusion or extensive tissue washing may reduce blood-associated absorption, whereas optimization of tissue disruption, buffer composition, processing time and temperature, and clarification conditions may improve recovery of active reporter and reduce its retention on insoluble debris.

The absolute calibration parameters and estimated LOD/LOQ values reported here are specific to male BALB/c mice, the tested sample-preparation protocol, assay composition and instrumental settings. The liver tissue-load experiment illustrates a general analytical trade-off but does not define a universal optimum for other organs. Similar behavior may occur in other tissues, although its magnitude will depend on organ-specific optical density, residual blood content, biochemical composition and extraction efficiency. In relatively transparent matrices, the increase in total reporter may outweigh the reduction in analytical response as tissue load increases, as observed for liver under the tested conditions. In more strongly absorbing matrices, attenuation may dominate, and increasing tissue input may provide little or no improvement in detectability. Tissue load should therefore be standardized and validated for each organ and experimental protocol.

### 3.3. Effect of Assay Environment on NanoLuc-Independent Furimazine Luminescence

The organ matrix experiments showed that the lysis buffer itself produced a substantial furimazine-dependent signal in the absence of NanoLuc. Buffer-associated substrate luminescence is not unique to the present assay: coelenterazine-dependent autoluminescence has previously been reported in detergent-containing lysis buffers [33,45]. We therefore examined the contribution of individual components of the standard lysis buffer to NanoLuc-independent furimazine luminescence. NP-40 was identified as the main contributor, whereas Tris-HCl, NaCl and glycerol produced substantially lower signals under matched conditions (Figure S11a). We then compared NP-40 with other commonly used nonionic detergents, including Triton X-100, Tween-20 and Tween-80. Detergent identity markedly affected furimazine-dependent background, with Tween-80 producing the lowest signal among the tested detergents (Figure S11b). The pronounced dependence on detergent identity suggests that substrate partitioning into detergent-associated microenvironments may contribute to the observed effect, although the underlying reaction mechanism was not directly investigated. However, detergent choice must also account for tissue disruption efficiency, protein extraction and preservation of NanoLuc activity; therefore, buffer optimization can improve assay performance but does not replace matrix-matched blank correction. Other common assay additives, including reducing agents, protease inhibitors and chelators, may also influence NanoLuc activity or furimazine-associated background and should therefore be kept consistent between samples and their corresponding blanks.

To separate protein- and serum-associated substrate background from the luminescence contribution of the lysis buffer, all subsequent experiments were performed in PBS, which produced negligible substrate-dependent luminescence under the tested conditions. Because organ blank signals broadly followed protein concentration, we next tested optically clearer serum-derived matrices. Furimazine produced detectable NanoLuc-independent luminescence in both fetal bovine serum (FBS) and mouse serum, and the signal decreased upon dilution (Figure 4a). The signals in bovine and mouse serum were comparable, indicating that this effect was not restricted to one serum source. Coelenterazine H (CTZ-h) generated substantially higher nonspecific luminescence than furimazine in the same matrices, by approximately one order of magnitude (Figure 4b). Thus, furimazine produces markedly lower background than CTZ-h, but it is not completely free of NanoLuc-independent luminescence in protein-containing matrices.

To evaluate whether major serum protein classes could contribute to this effect, we tested albumins and globulins using BSA and IgG as representative examples, with ovalbumin included as an unrelated protein control at matched molar concentrations. NanoLuc was included as an enzymatic positive control. Among the non-luciferase proteins, only BSA produced a signal clearly above the background, whereas IgG and ovalbumin showed minimal substrate-associated luminescence (Figure 4c). However, at the same nominal protein concentration, NanoLuc emitted approximately five orders of magnitude more light than BSA. Thus, albumin-supported furimazine luminescence is unlikely to affect measurements at high NanoLuc abundance, but it can become relevant at low reporter concentrations and should be accounted for using matrix-matched blanks.

BSA titration confirmed a concentration-dependent furimazine background over a broad range of albumin concentrations from 50 to 0.00064 g/L, with an approximately linear response over nearly three orders of magnitude (Figure 4d). Conversely, FMZ titration at fixed BSA concentration showed that albumin-supported luminescence increased with substrate concentration and did not reach a plateau within the tested range up to 200 µM FMZ (Figure 4e). These data indicate that NanoLuc-independent furimazine luminescence depends on both protein abundance and substrate availability.

Finally, we compared furimazine-dependent emission spectra generated in the presence of NanoLuc, purified BSA or FBS. NanoLuc produced the expected blue emission profile with an apparent maximum around 450–460 nm (Figure 4f) [19]. In contrast, albumin-dependent furimazine luminescence showed a short-wavelength spectrum with an apparent maximum at approximately 366 nm and only partial overlap with NanoLuc emission. Serum produced a more complex spectral profile, with a short-wavelength component resembling the BSA-associated signal and a broader component extending into the NanoLuc detection window. This indicates that albumin alone does not fully explain serum-associated furimazine luminescence. Other serum components or matrix-specific effects on substrate binding, oxidation chemistry, emitting-state stabilization or optical filtering may contribute to the observed signal.

During assay optimization, we also observed that furimazine handling can affect assay reproducibility. Furimazine stocks prepared in organic solvents such as DMSO or ethanol were relatively convenient for storage and dilution, but after contact with aqueous solutions the substrate became more prone to nonenzymatic oxidation. This effect is enhanced by prolonged exposure to light and air and is particularly noticeable when diluted working solutions are stored before use. Therefore, substrate working solutions should be prepared freshly, protected from unnecessary light and air exposure, and used within a fixed time window after dilution. In addition, kinetic acquisition with an AUC-based readout integrated signal differences over time, making subtle distinctions between weak reporter-derived and NanoLuc-independent signals more apparent than at a single endpoint.

## 4. Conclusions

This study shows that organ-derived matrices can influence ex vivo NanoLuc–furimazine measurements through differences in optical properties, NanoLuc-independent background and analytical response. Matrix-matched blank subtraction substantially improved low-concentration comparisons among the tested solid-organ homogenates, whereas matrix-specific calibration remained important for optically dense samples and for conversion of luminescence into reporter concentration. Detection and quantification limits reflected both calibration sensitivity and biological blank variability and therefore did not directly parallel raw signal attenuation.

Tissue load represented an additional determinant of assay performance. Increasing liver input altered optical properties, background luminescence and calibration sensitivity. However, in the pre-homogenization experiment, where NanoLuc input increased proportionally with tissue mass, the resulting increase in reporter-derived luminescence outweighed the reduction in analytical response observed in denser homogenates, thereby improving overall assay detectability under the tested conditions. These findings indicate that tissue load should be selected as a balance between the amount of reporter available for detection and matrix-dependent reduction in analytical response and should be standardized across samples.

NanoLuc-independent furimazine luminescence arising from lysis-buffer components and protein-containing samples further emphasizes the importance of matched blanks and consistent assay conditions. Although the purified NanoLuc spike-in model does not reproduce all variables present in delivery or gene-expression studies, it isolates analytical matrix effects that remain relevant whenever reporter activity is measured in tissue-derived lysates. Standardized sampling and homogenization, kinetic acquisition, matrix-matched background correction and matrix- or tissue-load-specific calibration may therefore improve the interpretation of weak ex vivo NanoLuc signals.

## Figures and Tables

**Figure 1:**
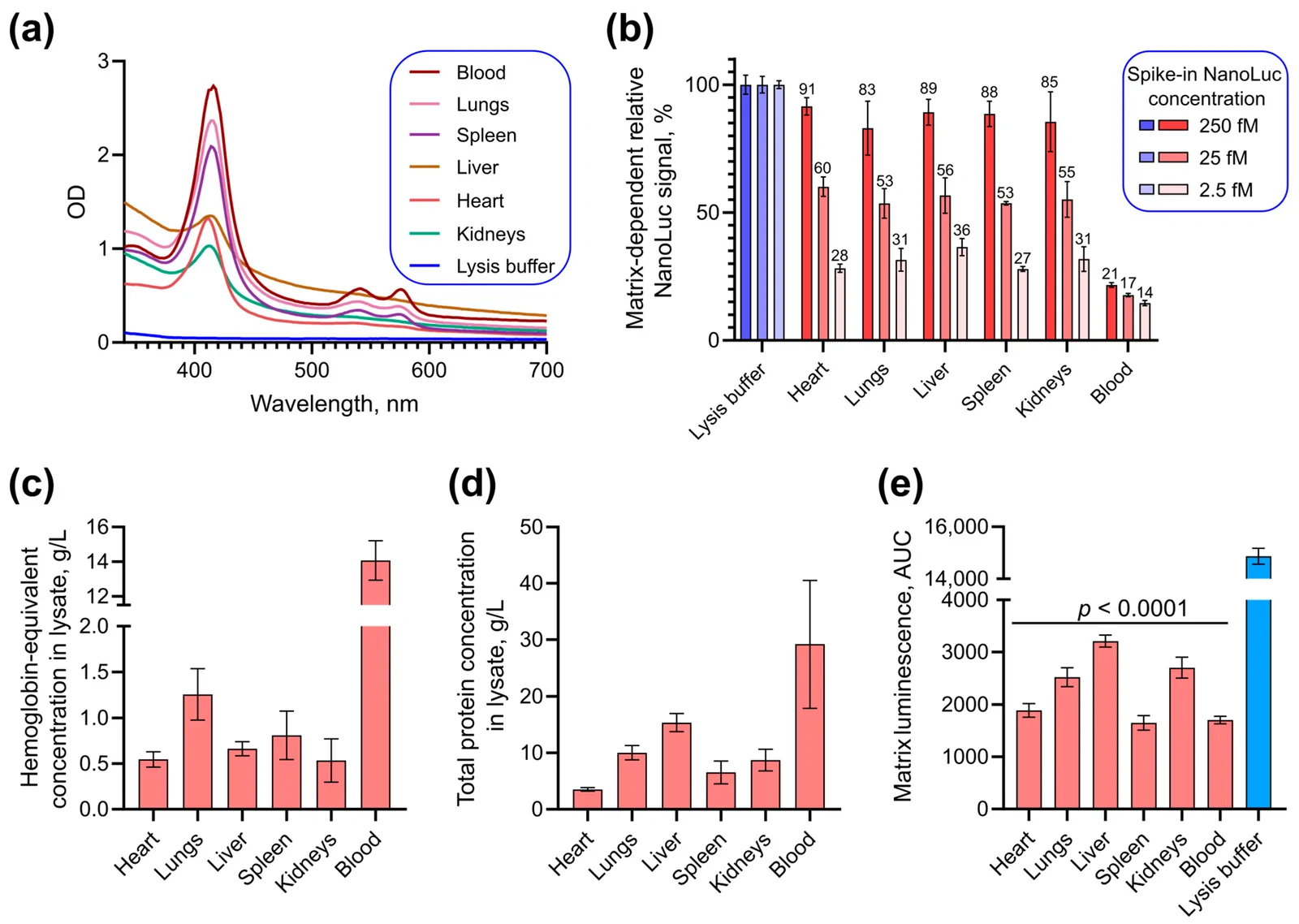
Effect of organ-derived matrices on NanoLuc detection. (**a**) Absorbance spectra of lysis buffer and mouse tissue matrices measured over the 340–700 nm range. The blood sample was additionally diluted 10-fold for spectral acquisition to avoid detector overflow. (**b**) Raw NanoLuc–furimazine AUC values in organ matrices expressed as a percentage of the corresponding NanoLuc signal in lysis buffer at three final spiked NanoLuc concentrations, without matrix-matched blank subtraction. The corresponding percentages are indicated above the bars. Blue bars indicate lysis buffer and red bars indicate organ matrices; shade intensity denotes the NanoLuc concentration. (**c**) A414-derived hemoglobin-equivalent absorbance metric used as an indicator of hemoglobin-associated optical burden in each matrix. (**d**) Total extracted protein concentration in organ lysate supernatants determined by BCA assay. (**e**) Matrix-associated furimazine background luminescence measured in the absence of NanoLuc. Data are shown as mean ± SD for *n* = 6 mice in panels (**b**–**d**) and *n* = 12 mice in panel (**e**). Differences in background luminescence among the biological matrices in panel (**e**), excluding lysis buffer, were assessed by RM one-way ANOVA.

**Figure 2:**
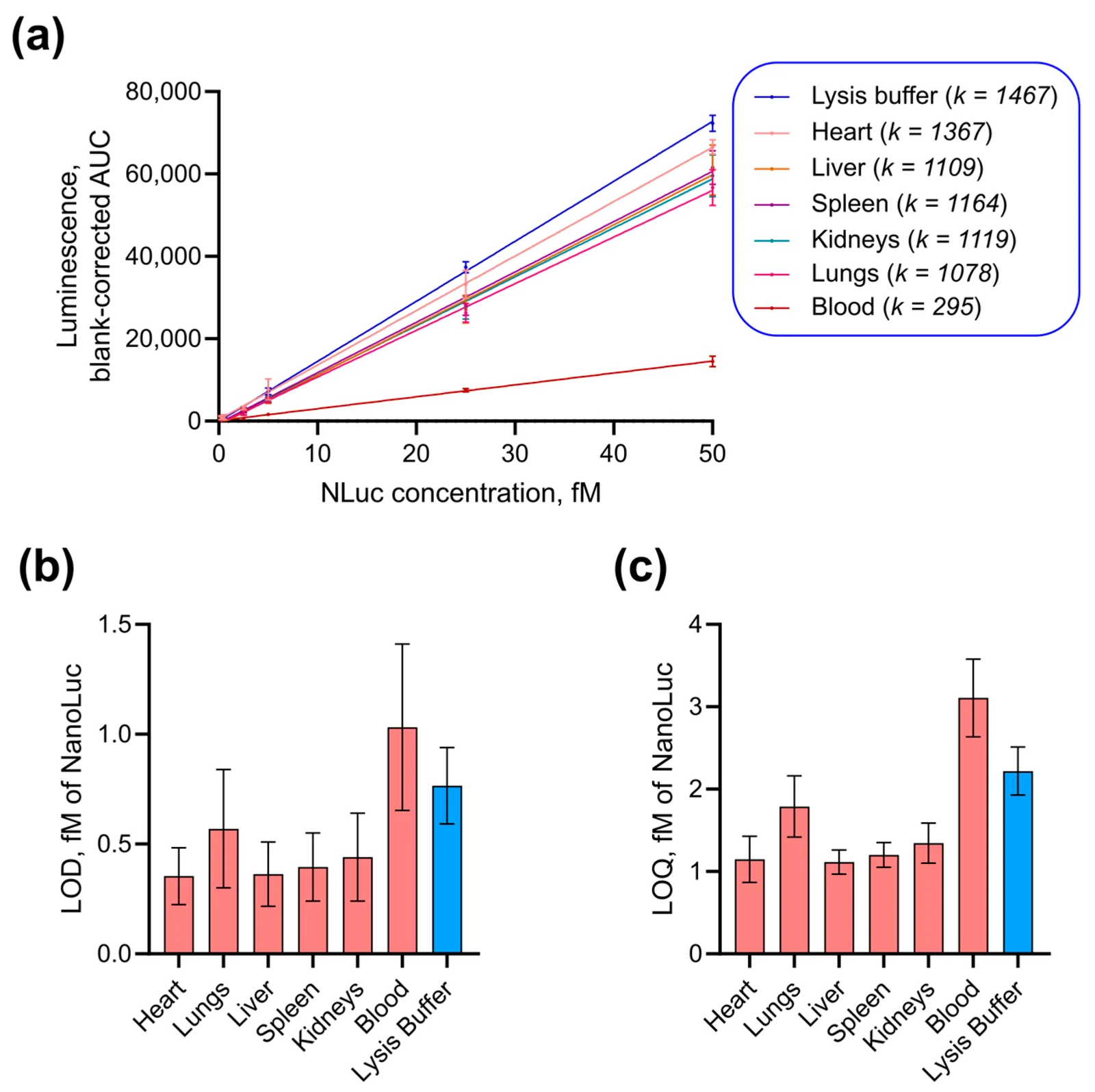
Matrix-specific NanoLuc calibration behavior and limits of detection (LOD) and quantification (LOQ) in organ homogenates. (**a**) Matrix-specific NanoLuc calibration curves generated by titrating purified NanoLuc into lysis buffer or organ homogenates in the presence of furimazine after subtraction of the corresponding matrix-matched blank. Values indicated as *k* in the legend correspond to the slopes of the linear calibration curves. Estimated matrix-specific limits of detection (**b**) and limits of quantification (**c**) of NanoLuc in organ homogenates calculated from biological blank distributions and matrix-specific calibration equations. Data are shown as mean ± SD, *n* = 6.

**Figure 3:**
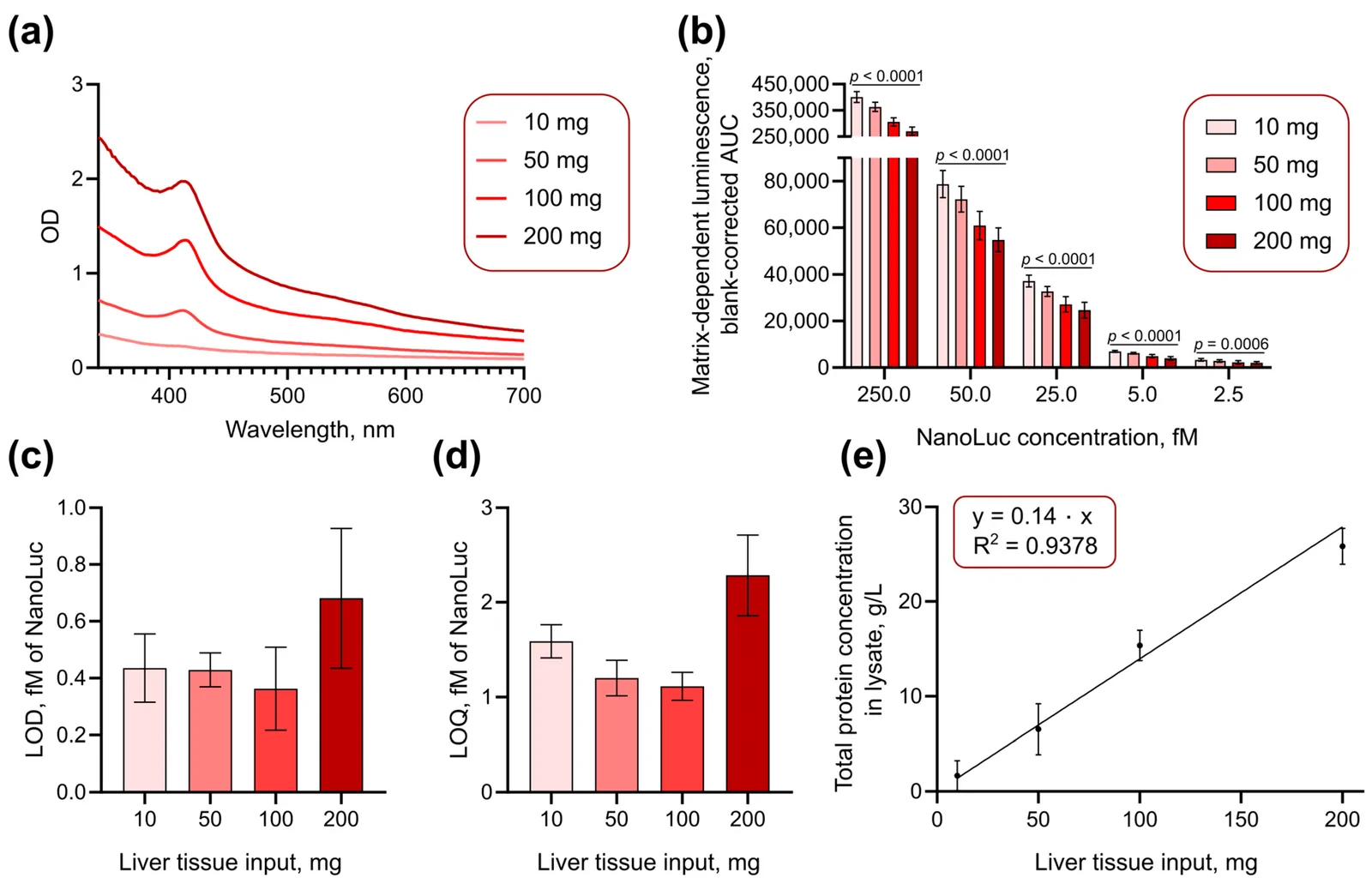
Effect of liver tissue load on NanoLuc–furimazine assay performance. (**a**) Absorbance spectra of liver homogenates prepared using 10, 50, 100 or 200 mg tissue per 1 mL lysis buffer. (**b**) Blank-corrected NanoLuc–furimazine response in liver homogenates with different tissue inputs at selected spiked NanoLuc concentrations. Differences among tissue loads were assessed separately at each NanoLuc concentration using RM one-way ANOVA. The resulting omnibus *p*-values were adjusted across concentrations using the Holm–Šídák method; adjusted *p*-values are shown. Estimated tissue-load-specific limits of detection (**c**) and limits of quantification (**d**) calculated from liver homogenate calibration curves and corresponding matrix-matched blanks. (**e**) Total protein concentration in liver lysate supernatants determined by BCA assay. Data are shown as mean ± SD, *n* = 6.

**Figure 4:**
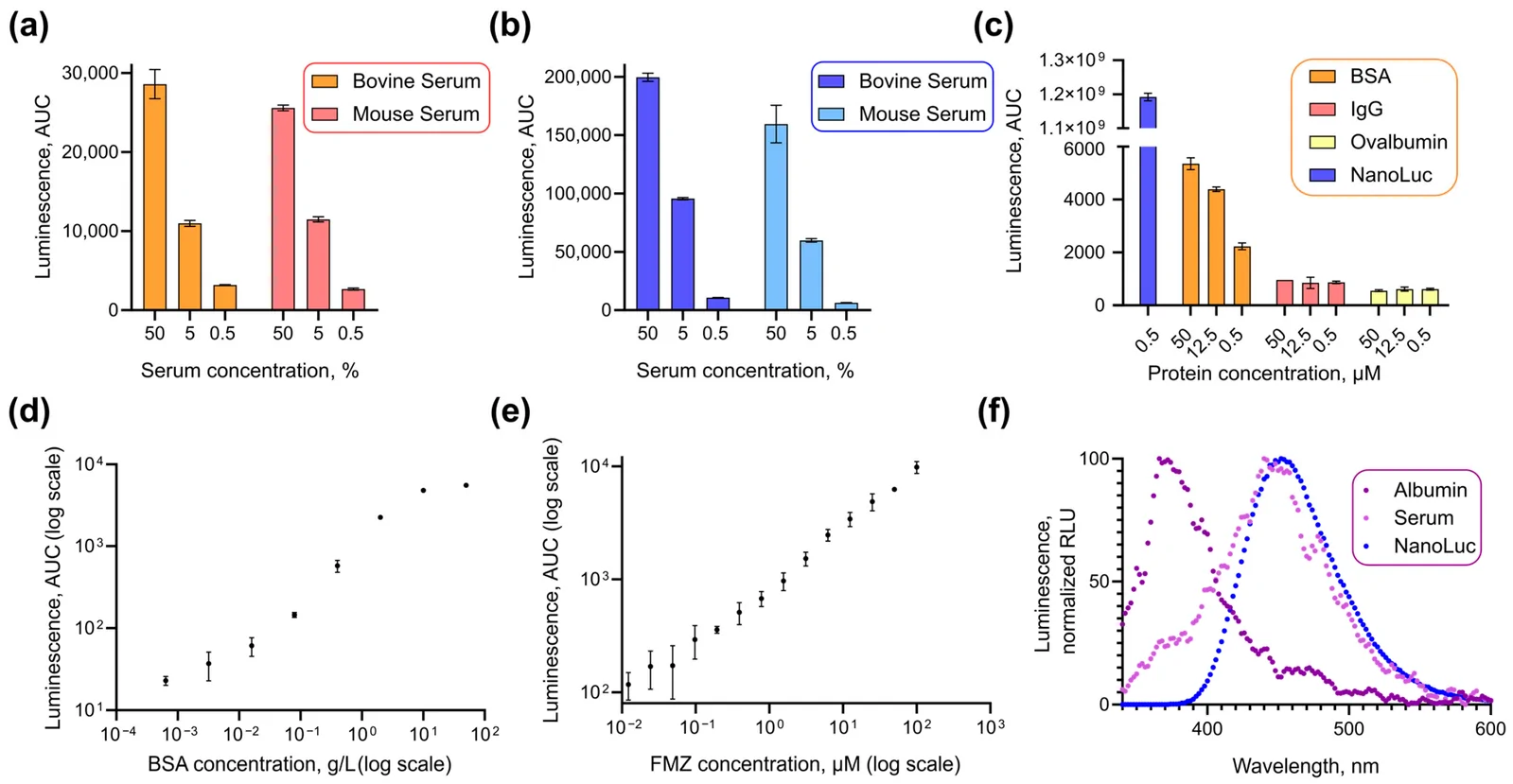
NanoLuc-independent furimazine luminescence in serum and protein matrices. (**a**,**b**) FMZ-dependent luminescence (**a**) and CTZ-h-dependent luminescence (**b**) in FBS and mouse serum. (**c**) Protein-dependent FMZ luminescence in the presence of purified BSA, IgG and ovalbumin at equimolar concentrations. NanoLuc was included as an enzymatic positive control at 0.5 µM. (**d**,**e**) Dependence of BSA-supported background luminescence on BSA concentration (**d**) and FMZ concentration (**e**). (**f**) Luminescence emission spectra of FMZ in the presence of BSA, FBS or NanoLuc. Data in panels (**a**–**e**) are shown as mean ± SD, *n* = 3 independent assay measurements. Logarithmic scales are used where indicated.

## Data Availability

The original contributions presented in this study are included in the article/Supplementary Material. Further inquiries can be directed to the corresponding authors.

## Supplementary Materials

The following supporting information can be downloaded at https://www.mdpi.com/article/10.3390/s26196135/s1: Figure S1: One-hour luminescence kinetics of NanoLuc and matrix-associated background; Figure S2: Hemoglobin calibration for estimation of hemoglobin-associated optical burden; Figure S3: Effect of BSA used for NanoLuc dilution on NanoLuc-independent furimazine luminescence; Figure S4: Uncorrected NanoLuc calibration curves in organ matrices; Table S1: Parameters of uncorrected NanoLuc calibration curves, matrix-matched blank signals, LOD and LOQ values in organ matrices; Figure S5: Representative weighted residual plots for matrix-specific NanoLuc calibration curves; Figure S6: Matrix-aware interpretation of NanoLuc signals in organ homogenates; Figure S7: Comparison of NanoLuc signal after post-lysis and pre-homogenization spike-in; Figure S8: Optical and background characteristics of liver homogenates prepared with different tissue inputs; Figure S9: Tissue-load-specific uncorrected NanoLuc calibration curves in liver homogenates; Table S2: Parameters of uncorrected NanoLuc calibration curves, matrix-matched blank signals, LOD and LOQ values in liver homogenates prepared using different tissue inputs; Figure S10: Pre-homogenization NanoLuc spike-in into liver samples with different tissue inputs; Figure S11: Effect of lysis buffer components and detergents on NanoLuc-independent furimazine luminescence; Figure S12: One-hour kinetics of NanoLuc-independent furimazine luminescence in protein-containing samples; Figure S13: Raw luminescence spectra of furimazine-containing samples.

## Abbreviations

The following abbreviations are used in this manuscript:

ATP	adenosine triphosphate
AUC	area under the curve
BCA	bicinchoninic acid
BSA	bovine serum albumin
CI	confidence interval
CTZ-h	coelenterazine h
DMSO	dimethyl sulfoxide
DNA	deoxyribonucleic acid
DTT	dithiothreitol
FBS	fetal bovine serum
FMZ	furimazine
IgG	immunoglobulin G
IPTG	isopropyl β-D-1-thiogalactopyranoside
LOD	limit of detection
LOQ	limit of quantification
mRNA	messenger ribonucleic acid
Ni^2+^-NTA	nickel–nitrilotriacetic acid
NP-40	Nonidet P-40 substitute
OD	optical density
PBS	phosphate-buffered saline
PMSF	phenylmethylsulfonyl fluoride
RLU	relative light units
RM	repeated-measures
SD	standard deviation
Tris	tris(hydroxymethyl)aminomethane
2×YT	double-strength yeast extract tryptone medium
