# Supplementary material for: Matrix Effects on the Quantification of NanoLuc Bioluminescence in Ex Vivo Organ Homogenates
Source: Sensors (Basel). 2026 Sep 28;26(19):6135. doi: 10.3390/s26196135 (PMC13667741; doi:10.3390/s26196135)
Supplement: Supplementary file 1 [file sensors-26-06135-s001.zip › sensors-4534447-supplementary.pdf]

Supplementary Information

Matrix effects on the quantification of NanoLuc bioluminescence in ex vivo organ homogenates

Roman O. Melikov [1,*], Maria O. Samokhina [1], Maria A. Yurchenko [1], Alexey V. Orlov [2], Maxim P. Nikitin [1,3,4,*]

[1] Moscow Center for Advanced Studies, 123592 Moscow, Russia

[2] Prokhorov General Physics Institute of the Russian Academy of Sciences, 119991 Moscow, Russia

[3] Department of Nanobiomedicine, Sirius University of Science and Technology, 354340 Sirius, Russia

[4] Shemyakin-Ovchinnikov Institute of Bioorganic Chemistry, Russian Academy of Sciences, 117997 Moscow, Russia

[*] Correspondence: r.o.melikov@gmail.com (R.O.M.); nikitin.mp@talantiuspeh.ru (M.P.N.)

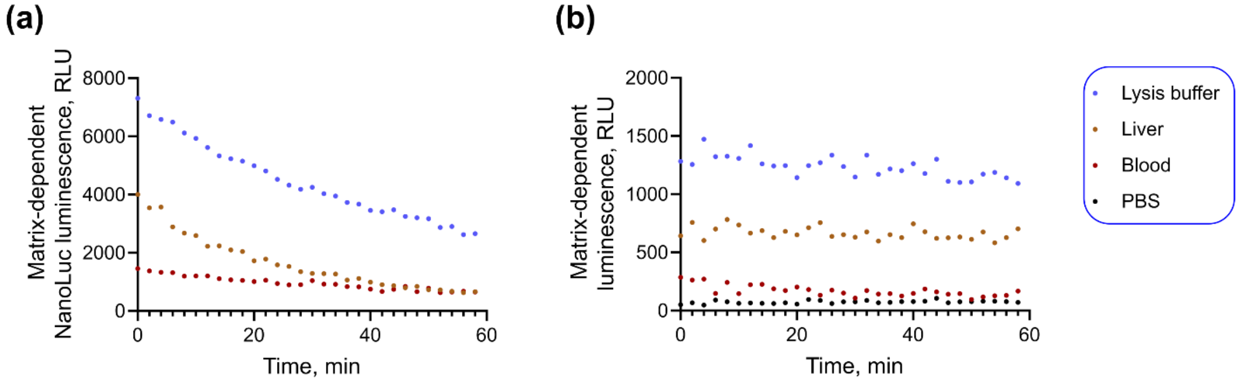

Supplementary Figure S1. One-hour luminescence kinetics of NanoLuc and matrix-associated background. (**a**) Representative one-hour luminescence kinetics of 25 fM NanoLuc measured in lysis buffer, liver lysate and blood lysate in the presence of 50 µM furimazine. The first 20 min of acquisition were used for the AUC-based analysis applied throughout the study. (**b**) NanoLuc-independent furimazine luminescence kinetics measured in lysis buffer, liver lysate and blood lysate, with PBS included as a negative control. These data illustrate the time-dependent behavior of enzymatic NanoLuc signal and substrate-associated background in different matrices.

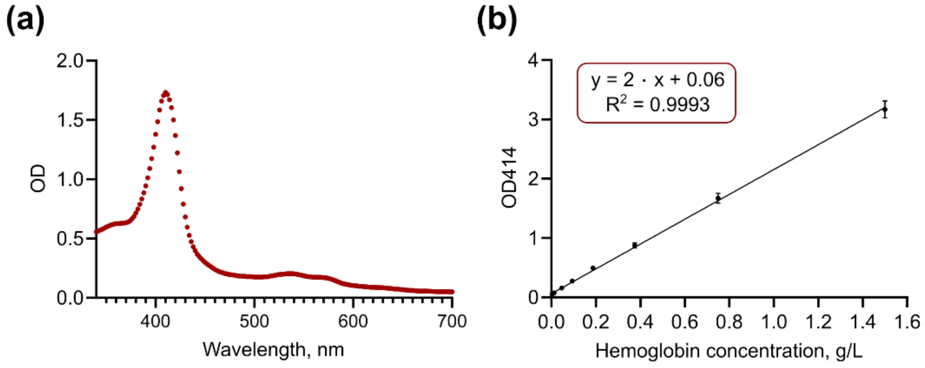

Supplementary Figure S2. Hemoglobin calibration for estimation of hemoglobin-associated optical burden. (**a**) Absorbance spectrum of hemoglobin standard solution measured at 0.75 g/L. (**b**) Hemoglobin calibration curve based on absorbance at 414 nm. The A414-derived hemoglobin-equivalent metric was calculated as: $c = (\text{OD414} - 0.06) / 2 \times$ dilution factor, where c is expressed in g/L. The dilution factor was 1 for organ lysates and 10 for blood lysate.

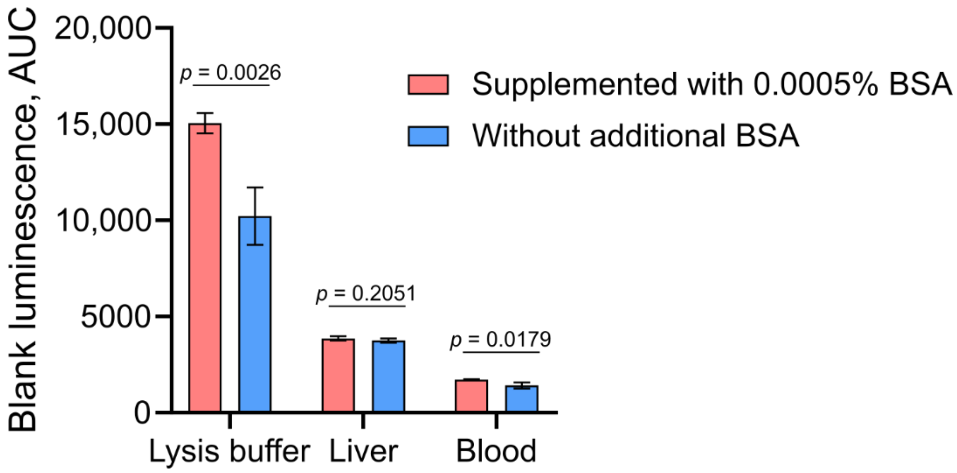

Supplementary Figure S3. Effect of BSA used for NanoLuc dilution on NanoLuc-independent furimazine luminescence. Identical volumes of PBS containing 0.01% BSA or PBS alone were added to lysis buffer, liver or blood lysates before furimazine addition. The final BSA concentration was 0.0005%, matching that in NanoLuc-containing samples and matrix-matched blanks. BSA increased luminescence most strongly in lysis buffer, which retained the highest blank signal even without BSA. Data are shown as mean ± SD, $n = 4$. Within each matrix, conditions with and without BSA were compared using Welch's t-test with Holm–Šídák correction for multiple comparisons; adjusted p-values are shown.

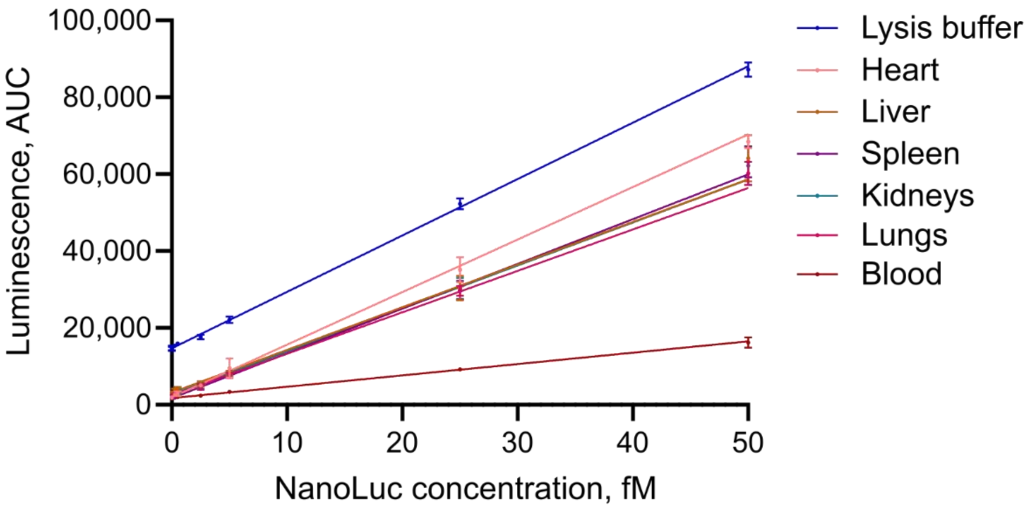

Supplementary Figure S4. Uncorrected NanoLuc calibration curves in organ matrices. Calibration curves were generated by titrating purified NanoLuc into lysis buffer or organ-derived matrices in the presence of furimazine, without subtraction of the corresponding matrix-matched blank. Data are shown as mean ± SD, $n = 6$.

Supplementary Table S1. Parameters of uncorrected NanoLuc calibration curves, matrix-matched blank signals, estimated LOD and LOQ values in organ matrices.

Matrix	Heart	Lungs	Liver	Spleen	Kidneys	Blood	Lysis buffer
**Slope [95% CI], AUC/fM**	1367 [1269 – 1466]	1078 [1022 – 1133]	1109 [1041 – 1177]	1164 [1102 – 1227]	1119 [1051 – 1187]	295.3 [281.9 – 308.6]	1467 [1428 – 1507]
**Y-intercept [95% CI], AUC**	1958 [1815 – 2101]	2506 [2389 – 2622]	3180 [3014 – 3346]	1705 [1613 – 1798]	2674 [2529 – 2820]	1742 [1688 – 1796]	14696 [14468 – 14924]
**R^2**	0.9828	0.989	0.9822	0.9909	0.9875	0.9886	0.998
**Blank mean ± SD, AUC**	1891 ± 131	2521 ± 179	3211 ± 115	1652 ± 140	2702 ± 201	1705 ± 71	14872 ± 308
**LOD [95% CI], fM**	0.35 [0.22 – 0.49]	0.57 [0.29 – 0.85]	0.36 [0.18 – 0.54]	0.4 [0.23 – 0.56]	0.44 [0.23 – 0.65]	1.03 [0.63 – 1.43]	0.77 [0.58 – 0.95]
**LOQ [95% CI], fM**	1.15 [0.85 – 1.44]	1.79 [1.4 – 2.18]	1.11 [0.96 – 1.27]	1.2 [1.04 – 1.36]	1.35 [1.09 – 1.6]	3.11 [2.61 – 3.6]	2.22 [1.91 – 2.53]

Calibration curves were generated using organ matrices obtained from six independent mice and were fitted over the final NanoLuc concentration range of 0–50 fM without subtraction of the matrix-matched blank. Technical replicates were averaged before fitting. Matrix-matched blank values were obtained from 12 independent mice and are presented as mean ± SD. Calibration slope and Y-intercept are presented with 95% confidence intervals. LOD and LOQ were calculated from the corresponding biological blank distributions and matrix-specific calibration equations generated for each of the six independent individual calibration curves; the resulting values were then averaged and are presented as mean values with 95% confidence intervals.

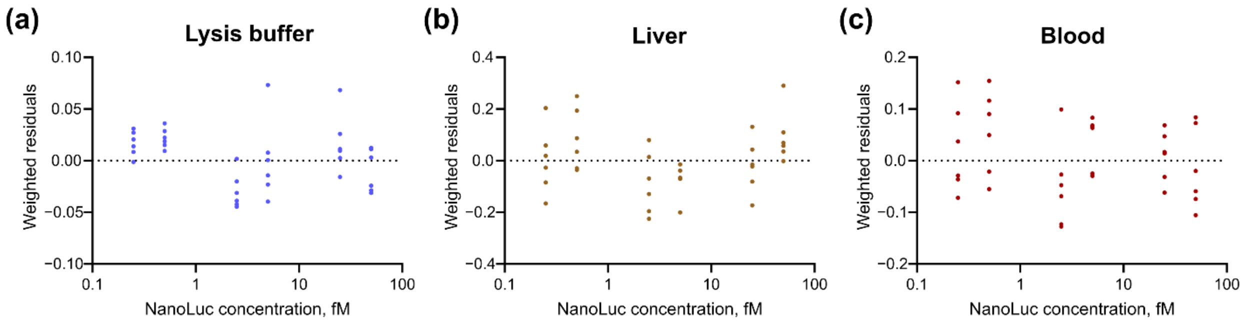

Supplementary Figure S5. Representative weighted residual plots for matrix-specific NanoLuc calibration curves. Weighted residuals are shown for lysis buffer (**a**), liver homogenate (**b**) and blood lysate (c) after fitting raw AUC versus NanoLuc concentration by weighted least-squares regression using $1/y^2$ weighting. The residuals are distributed around zero without a pronounced increase in dispersion at the higher response values used for calibration.

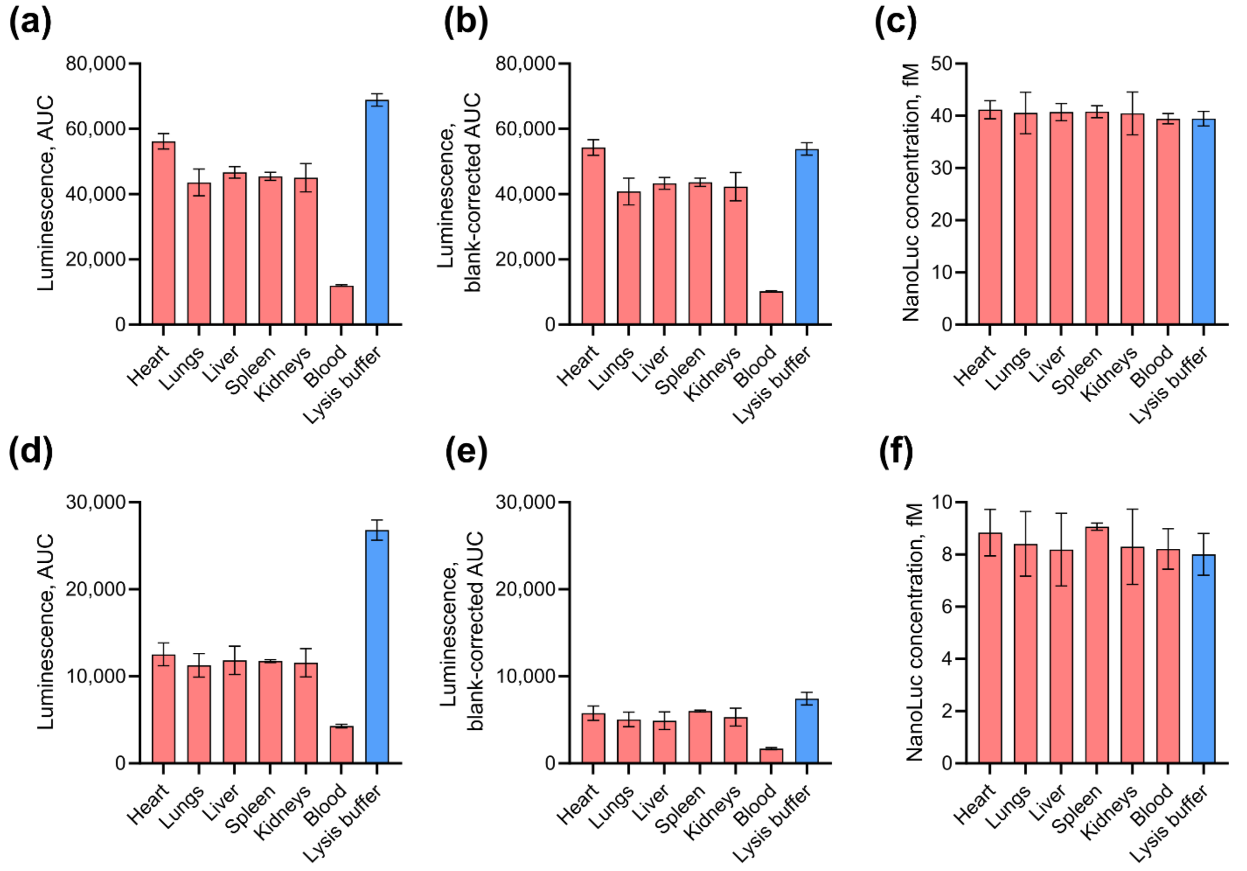

Supplementary Figure S6. Matrix-aware interpretation of NanoLuc signals in organ homogenates. (**a–c**) Example analysis for 40 fM NanoLuc spiked into organ matrices. Raw luminescence signals were first compared directly between matrices and lysis buffer (**a**), then corrected by subtracting the corresponding matrix-matched substrate blank (**b**), and finally converted to NanoLuc concentration equivalents using matrix-specific calibration curves (**c**). (**d–f**) The same analysis for 8 fM spiked NanoLuc. Matrix-matched blank subtraction reduced substrate-associated background, but did not fully normalize low-concentration NanoLuc signals between all tested tissues. Calibration-based estimates improved quantitative comparability across matrices and enabled direct comparison with optically dense matrices, such as blood, where raw luminescence values are strongly affected by light absorption. Data are shown as mean ± SD, $n = 3$.

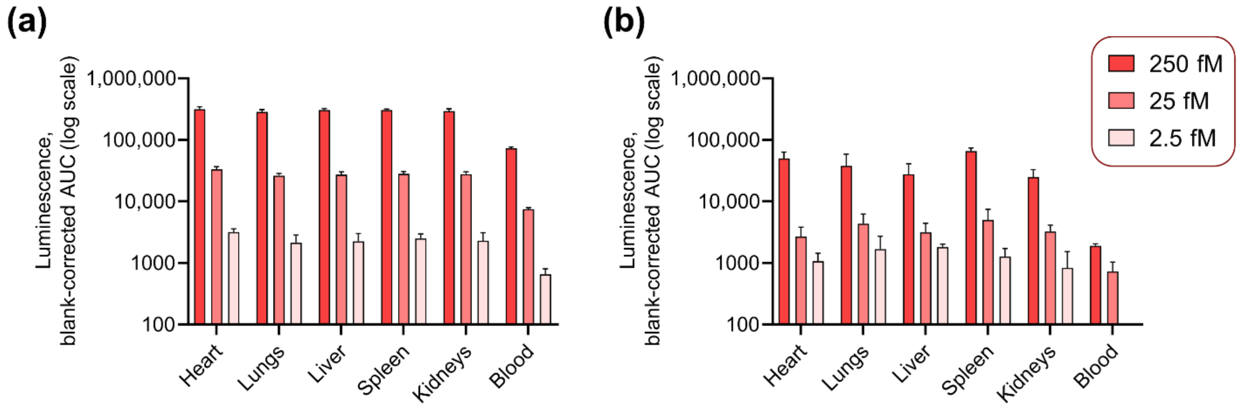

Supplementary Figure S7. Comparison of NanoLuc signal after post-lysis and pre-homogenization spike-in. NanoLuc-furimazine luminescence measured in organ lysates after addition of purified NanoLuc to clarified lysates (**a**) or after addition of purified NanoLuc to organ samples before homogenization (**b**). NanoLuc was tested at final nominal concentrations of 250, 25 and 2.5 fM. For blood at 2.5 fM after pre-homogenization spike-in, the matrix-matched blank-corrected AUC was non-positive and is therefore not displayed. The comparison illustrates the additional variability and potential signal loss introduced during tissue processing before clarification. Data are shown as mean ± SD, $n = 3$.

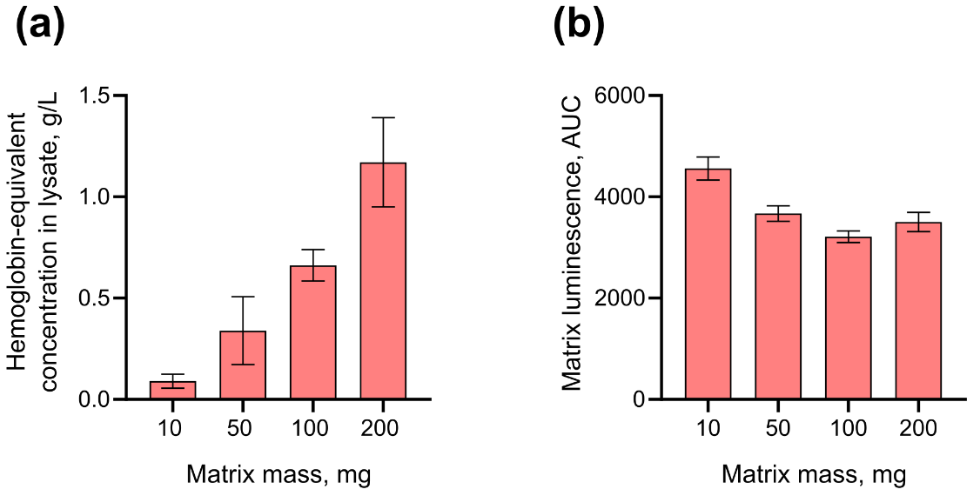

Supplementary Figure S8. Optical and background characteristics of liver homogenates prepared with different tissue inputs. (**a**) Hemoglobin-equivalent absorbance metric derived from the A414 calibration and used as an indicator of hemoglobin-associated optical burden in liver homogenates prepared using 10, 50, 100 or 200 mg tissue per 1 mL lysis buffer. (**b**) Matrix-associated furimazine background luminescence measured in the absence of NanoLuc in the liver homogenates. Data are shown as mean ± SD, $n = 3$ for panel (**a**) and $n = 12$ for panel (**b**).

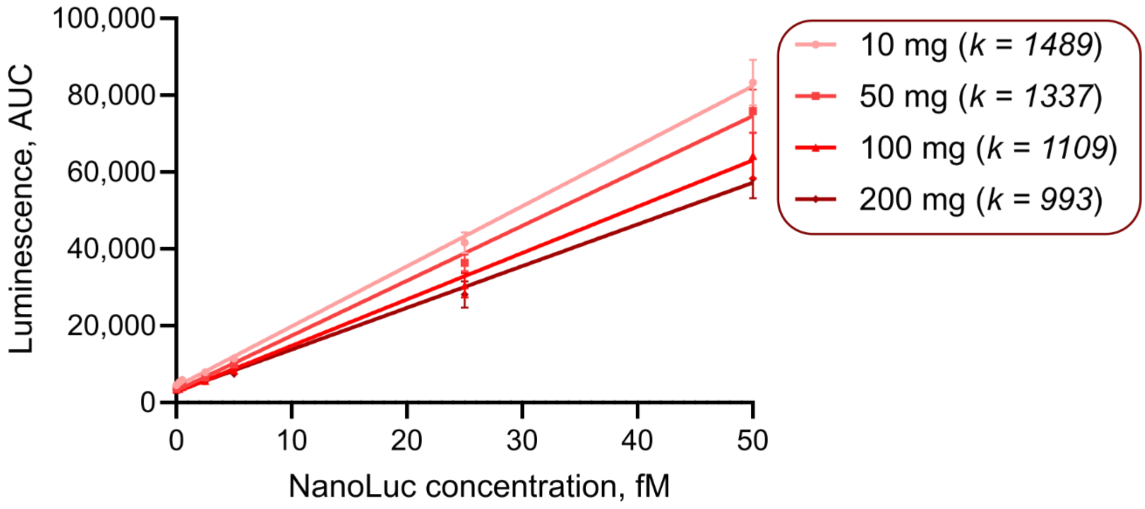

Supplementary Figure S9. Tissue-load-specific uncorrected NanoLuc calibration curves in liver homogenates. Calibration curves were generated by titrating purified NanoLuc into liver homogenates prepared using 10, 50, 100 or 200 mg tissue per 1 mL lysis buffer in the presence of furimazine without subtraction of the corresponding matrix-matched blanks. Values indicated as *k* in the legend correspond to the slopes of the linear calibration curves. Data are shown as mean ± SD, *n* = 6.

Supplementary Table S2. Parameters of uncorrected NanoLuc calibration curves, matrix-matched blank signals, estimated LOD and LOQ values in liver homogenates prepared using different tissue inputs.

Liver tissue input	10 mg	50 mg	100 mg	200 mg
**Slope [95% CI], AUC/fM**	1489 [1421 – 1557]	1337 [1284 – 1390]	1109 [1041 – 1177]	992.5 [931.8 – 1053]
**Y-intercept [95% CI], AUC**	4615 [4439 – 4790]	3656 [3531 – 3782]	3180 [3014 – 3346]	3476 [3305 – 3648]
**R^2**	0.9923	0.9912	0.9822	0.9850
**Blank mean ± SD, AUC**	4560 ± 226.5	3671 ± 152.6	3211 ± 115	3500 ± 189.9
**LOD [95% CI], fM**	0.44 [0.29 – 0.58]	0.43 [0.36 – 0.5]	0.36 [0.18 – 0.54]	0.68 [0.38 – 0.99]
**LOQ [95% CI], fM**	1.59 [1.4 – 1.77]	1.2 [1 – 1.4]	1.11 [0.96 – 1.27]	2.29 [1.84 – 2.73]

Calibration curves were generated using liver homogenates obtained from six independent mice and were fitted over the final NanoLuc concentration range of 0–50 fM without subtraction of the corresponding tissue-load-matched blank. Technical replicates were averaged before fitting. Tissue-load-matched blank values were obtained from 12 independent mice and are presented as mean ± SD. Calibration slope and Y-intercept are presented with 95% confidence intervals. LOD and LOQ were calculated from the corresponding biological blank distributions and tissue-load-specific calibration equations generated for each of the six independent individual calibration curves; the resulting values were then averaged and are presented as mean values with 95% confidence intervals.

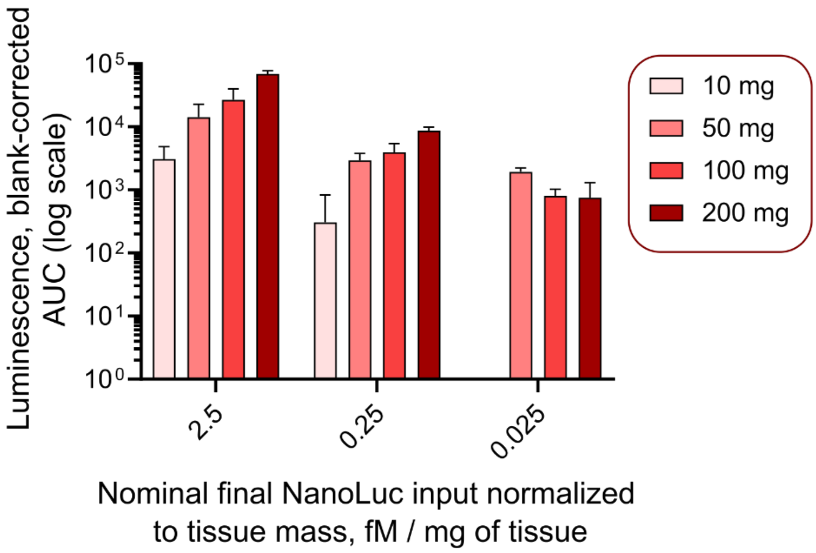

Supplementary Figure S10. Pre-homogenization NanoLuc spike-in into liver samples with different tissue inputs. Purified NanoLuc was added to liver samples before homogenization, with the total amount of NanoLuc scaled proportionally to tissue mass. Liver inputs of 10, 50, 100 and 200 mg per 1 mL lysis buffer were tested. This experiment models a situation in which reporter activity is distributed throughout the tissue and a larger tissue fragment contains a proportionally larger total amount of reporter. For the 10-mg sample at the lowest NanoLuc input level, the signal after blank subtraction was non-positive and was therefore considered below detection and could not be displayed on the logarithmic scale. Data are shown as mean ± SD, $n = 3$.

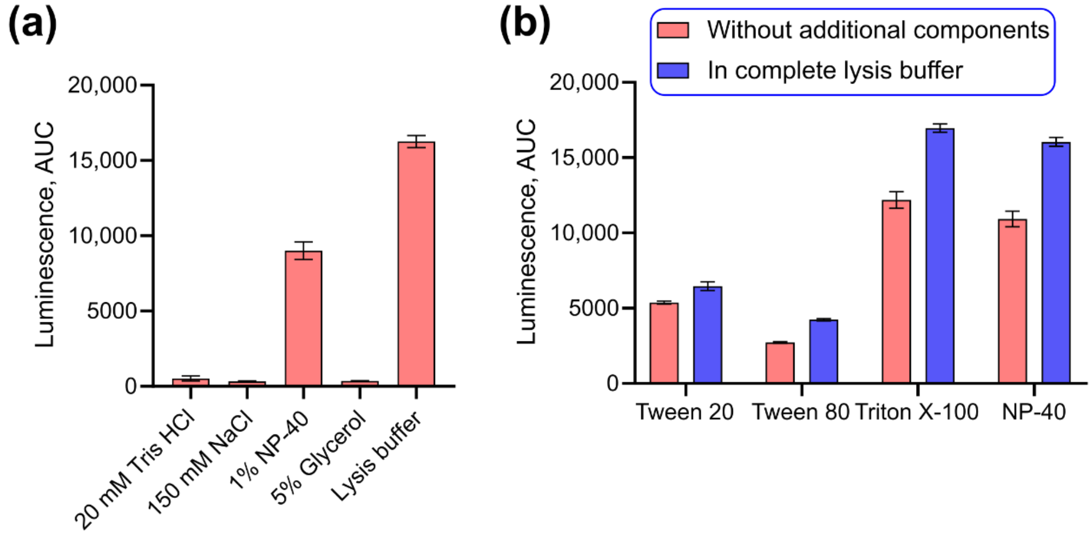

Supplementary Figure S11. Effect of lysis buffer components and detergents on NanoLuc-independent furimazine luminescence. (**a**) Furimazine-dependent luminescence measured in individual components of the standard lysis buffer and in complete lysis buffer. NP-40 was the main contributor to substrate-dependent background under these conditions. (**b**) Comparison of furimazine-dependent background in buffers containing different nonionic detergents, including NP-40, Triton X-100, Tween-20 and Tween-80. Each detergent was tested at 1% either without the remaining buffer components or in the complete lysis-buffer composition. Data are shown as mean ± SD, $n = 3$.

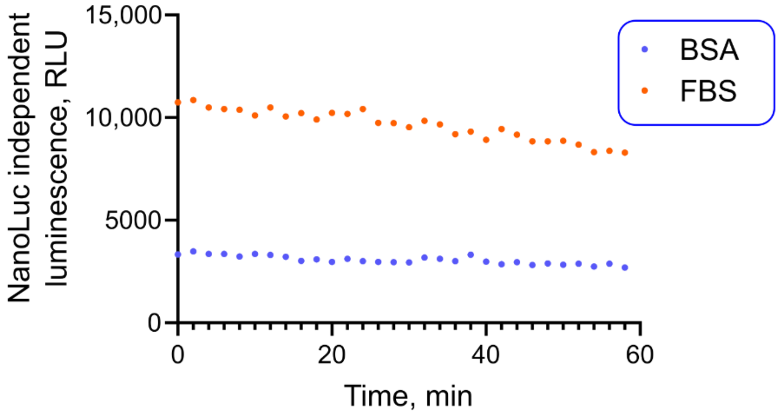

Supplementary Figure S12. One-hour kinetics of NanoLuc-independent furimazine luminescence in protein-containing samples. One-hour luminescence kinetics of furimazine-dependent background measured in the presence of 50 g/L BSA or 50% FBS at a final furimazine concentration of 50 µM and in the absence of NanoLuc. These data illustrate the time-dependent development of protein- and serum-associated substrate background during prolonged acquisition.

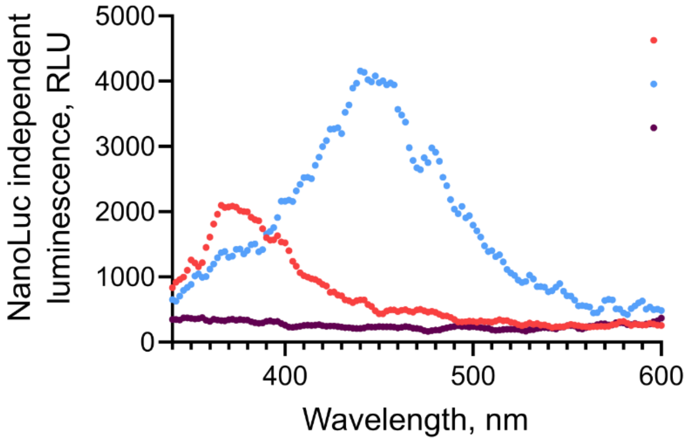

Supplementary Figure S13. Raw luminescence spectra of furimazine-containing samples. Raw, non-normalized luminescence spectra of samples containing 50 g/L BSA, 50% FBS or PBS at a final furimazine concentration of 50 µM. PBS with furimazine was included as a substrate/instrument control. These spectra complement the normalized spectral comparison and show the absolute signal levels before background correction or normalization.
